# Colloid and Interface Science for Understanding Microplastics and Developing Remediation Strategies

**DOI:** 10.1021/acs.langmuir.4c03856

**Published:** 2025-02-14

**Authors:** Ahmed Al Harraq, Philip J. Brahana, Bhuvnesh Bharti

**Affiliations:** † Joseph Henry Laboratories, Princeton University, Princeton, New Jersey 08544, United States; ‡ Cain Department of Chemical Engineering, Louisiana State University, Baton Rouge, Louisiana 70803, United States

## Abstract

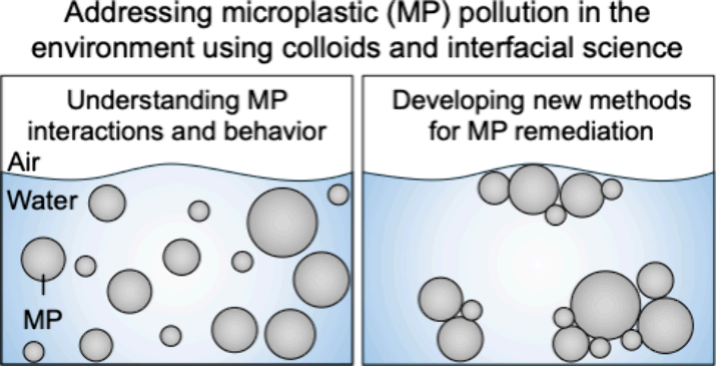

Microplastics (MPs) originate from industrial production of <1 mm polymeric particles and from the progressive breakdown of larger plastic debris. Their environmental behavior is governed by their interfacial properties, which dominate due to their small size. This Perspective highlights the complex surface chemistry of MPs under environmental stressors and discusses how physical attributes like shape and roughness could influence their fate. We further identify wastewater treatment plants (WWTPs) as critical hotspots for MP accumulation, where the MPs are inadvertently transferred to sewage sludge and reintroduced into the environment. We emphasize the potential of colloid and interfacial science not only to improve our fundamental understanding of MPs but also to advance mitigation strategies in hotspots such as WWTPs.

## 1 Introduction

**Figure 1:**
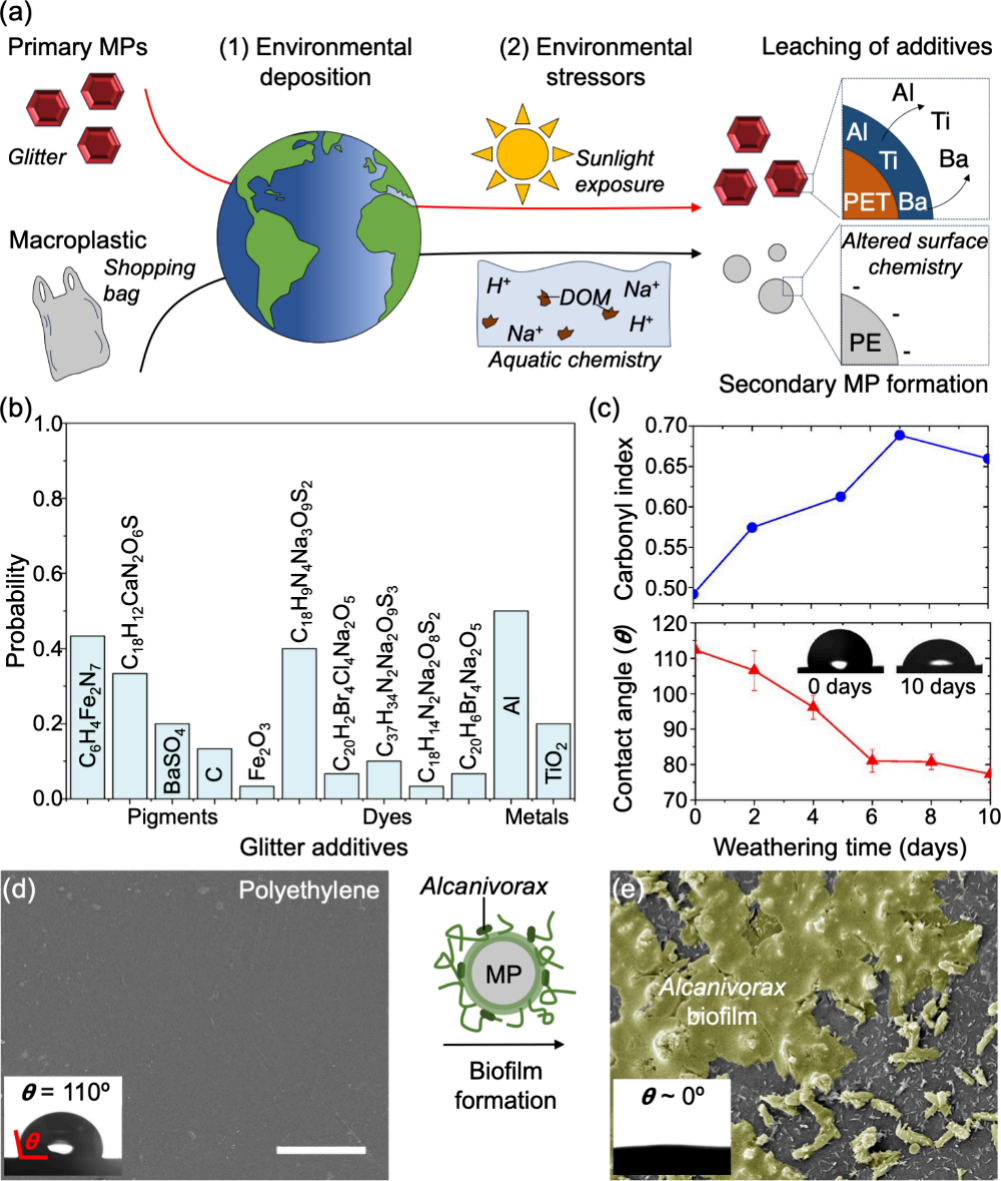
Effective interfacial chemistry of MPs. (a) Schematic describing the introduction of primary MPs and formation of secondary MPs in the environment, with associated dynamic changes in the surface chemistries as a result of weathering, and the adsorption of ions and dissolved organic matter (DOM). (b) Bar plot showing the probability of identifying specific chemical additives in commercial glitter from a survey of 36 different varieties examined via combined scanning electron microscopy and energy-dispersive X-ray spectroscopy (SEM-EDS). Results are grouped by additive type from values obtained by Najjar et al.^56^ (c) Carbonyl index and water contact angle of PE MPs as a function of weathering time, taken from ref (9). The carbonyl index is calculated from the Fourier transform infrared (FTIR) spectra as the ratio of the areas under the specified carbonyl and methylene bands, providing insight into the degree of photo-oxidation occurring to the PE MPs. Inset images are sessile water droplets on an unweathered (0 days) and weathered (10 days) PE substrate. (d and e) Scanning electron microscopy (SEM) images of a PE sheet before and after biofilm formation, respectively, by the marine bacterium *Alcanivorax borkumensis*. The biofilm in panel e is false colored in green for improved visualization. The scale bar in panel d is 5 μm. The insets of both panels d and e are the images of the water contact angle on the respective surface. Panel c is reproduced from ref (9). Available under a CC BY 4.0 license. Copyright 2022 Bhuvnesh Bharti.

Microplastics (MPs) are polymeric particles smaller than 1 mm that are increasingly being detected in many environments across the globe, from ocean bottoms to mountain peaks.^1^ Intentionally manufactured MPs such as microbeads in personal care products and industrial abrasives are referred to as primary MPs.^2−4^ Conversely, secondary MPs result from the degradation of larger plastic items such as plastic bags and bottles (Figure 1a).^5^ While inherently associated with the wider plastics’ environmental crisis, MPs differ significantly from larger plastic debris. This is because, unlike bulk plastics, the behavior of MPs is dominated by interfacial properties and chemistry, which impact their transport, aggregation, and potential to act as carriers for other pollutants.^6^ These characteristics differentiate MPs from both macroscale and molecular-scale kinds of pollutants, posing unique challenges in understanding their environmental fate and devising remediation strategies.^7^ In earlier work, we argued that the nanometer to millimeter length scales associated with this environmental issue underscored the need for a colloid science perspective in the fundamental study of MPs.^6^ Here, we explore how these principles can be applied toward mitigating the environmental burden of MPs.

Several key questions currently drive research on MPs, from the impact of environmental stressors on the surface properties^8−10^ to the synergistic effects on transport and aggregation caused by molecular adsorption on the surface of MPs.^11,12^ Further, the interaction of MPs with biota is of increasing relevance to compile risk assessments by determining the mechanisms and likelihood for biological uptake of the pollutants and their spreading through the food chain.^13,14^ Much ongoing effort is aimed at developing standardized analytical methods.^15^ These include the quantitative detection and characterization of MPs to differentiate between polymeric particles and natural colloids such as clay and biomass-derived particles (wool, hair, etc.) in real environments.^16^ In parallel to addressing the mechanistic questions and the methodological issues in MPs research, there is also increasing interest in devising remediation strategies to remove MPs and mitigate their potential ecotoxicity and adverse health effects.^17−19^ In this context, it must be noted that any mitigation effort will not effectively address the impacts of MPs without policy and societal changes.^20,21^

While various methods for MP removal are being developed at the laboratory scale,^22,23^ it is important to recognize that large-scale removal of MPs from the environment is inherently unsustainable. The continuous introduction of MPs in large “sinks” where they spontaneously mix and spread corresponds to an increase in disorder trending toward a state of maximum entropy. This dispersal into expansive environments such as oceans and soil makes the reversal of this entropic increase energetically demanding. Mitigation and removal efforts should focus on “hotspots” where MPs either accumulate or originate. These include household and industrial sources, as well as accumulation points such as drinking water and wastewater treatment plants (WWTPs).^24^ The WWTPs are of particular interest, as they not only collect large amounts of MPs from various sources but can also inadvertently generate MPs through breakdown of larger plastic debris throughout processing.^25,26^ Despite their potential role in mitigating plastic pollution, current WWTPs often lack unit operations that separate MPs from other types of sediment. Thus, MPs flowing into WWTPs often re-enter the environment via the municipal sludge that is output by the plant.^27^ Nevertheless, WWTPs offer a unique technical setting for introducing new removal techniques that leverage principles of colloid and interfacial science. In this Perspective, we will outline key interfacial and colloidal properties of MPs that require further investigation and discuss the potential for implementing targeted removal strategies in WWTPs. In conclusion, we provide an outlook with a summary of key research directions to encourage contributions from the colloid and interface science community.

## 2 Interfacial Chemistry of Microplastics

The surface chemistry of MPs governs the key properties such as wettability and adsorption capacity of MPs, which in turn determine their environmental fate and toxicity. Understanding these chemistries is far from trivial due to the complex manufactured composition of MPs, which often includes additives like plasticizers, flame retardants, dyes and stabilizers.^28^ Upon entering the environment, dynamic changes occur to the surface chemistry of MPs under the influence of stressors such as sunlight exposure,^9,29^ mechanical wear^30,31^ and biological activity.^8,32^ These stressors can cause leaching of additives and chemical transformation of the polymers, altering surface properties over time.^33^ For instance, primary MPs like glitter,^34^ originating from cosmetic, textile, and coating industries, are composed mainly of polyethylene terephthalate (PET) and contain additives such as pigments for coloration and heavy metals to achieve high reflectivity (Figure 1b). Over time, environmental weathering processes can cause the leaching of these additives, altering the surface chemistry of the particles.^35^ Similarly, secondary MPs experience significant changes in their surface chemistry due to the stresses involved in their formation. Such a dynamic nature of MPs surface chemistry complicates efforts to predict their behavior and track their environmental fate. There is extensive literature focusing on understanding the mechanisms of MPs formation,^36^ but key questions remain on the influence of additives on the kinetics of surface chemistry evolution. The interplay of multiple environmental stressors including both abiotic and biotic kinds, is a critical area of investigation requiring a combination of controlled lab-based efforts and field studies in real environments.

Exposure to environmental stressors profoundly alters the surface chemistry of MPs, even though most commercial plastics are engineered to be chemically inert with low surface energy and neutral charge. For example, we recently showed that exposure of polyethylene (PE) MPs to simulated sunlight promoted the formation of carboxyl surface groups, which correspondingly reduced the water contact angle (Figure 1c).^9^ Photo-oxidation increased the wettability of the particles and imparted them with a net negative surface charge. These changes, occurring within 10 days of accelerated weathering and well before any observable mechanical breakdown, increased the dispersibility of these otherwise hydrophobic MPs while increasing their surface uptake of common pollutant molecules such as lead ions. Efforts in correlating the changes in surface chemistry with the underlying molecular structure of the MPs are complicated by the presence of additives in commercial plastics. For example, a recent study found that brominated flame retardants in polystyrene films significantly alter both the rate and mechanism of photooxidation compared to films composed of pure polystyrene.^37^ However, the influence of these and other additives on the interfacial properties of MPs remains largely unclear. Addressing these knowledge gaps requires a combination of controlled laboratory studies and field research to investigate the interplay of multiple environmental stressors, including abiotic and biotic, on MPs weathering and lifecycle.

The surface chemistries of MPs and the changes they undergo over time must be contextualized to their surrounding environment. In fact, the coupling of surface and solution chemistry results in changes in the aggregation and transport of MPs.^38−40^ One area of particular interest is the interplay between stressor-induced chemical change of MPs surfaces and the adsorption of external molecules or formation of biofilms which in turn can modify the interfacial properties of the particles.^8,9,41−43^ For example, we recently showed how biofilms of *Alcanivorax borkumensis* on PE surfaces increase the wettability and sinking of MPs^8^ (Figure 1d-e). Specifically, the formation of biofilm was accompanied by the production of biosurfactants that induced a hydrophobic-to-hydrophilic transition of the MPs surface, i.e., increasing water wettability, thus promoting sinking. Adsorption of molecules/ions onto MPs and their aggregation are key to the risk assessment of MPs as vehicles for other pollutants such as heavy metal ions, perfluoroalkyl substances, as well as engineered nanomaterials. Numerous studies invoke Derjaguin–Landau–Verwey–Overbeek (DLVO) theory while documenting the aggregation of polystyrene MPs with increasing ionic strength.^44,45^ However, DLVO theory often oversimplifies the complexity of MPs interactions in natural environments.^46^ Key questions about the roles of dissolved organic matter (DOM), salinity gradients, and the propensity of MPs to form heteroaggregates with other particles and cells remain. Such knowledge gaps highlight the need to adopt extended DLVO theories that have better predictive value for colloids and interfaces with complex interaction potentials.^47^ The interaction of MPs with biological interfaces is a topic of increasing relevance to the assessment of health risks associated with these pollutants.^48^ Existing knowledge of bionano interfacial phenomena can inform predictions and experimental testing with colloidal MPs.^49^ For example, the potential for protein corona formation recently investigated by Schvartz et al. and its subsequent effect in cellular uptake and immune response.^50^

The existing knowledge of the surface chemistry of environmental colloids such as clays provides useful insight to address the many questions regarding MPs. For example, clay colloids are aluminosilicate particles with layered structures that are naturally abundant and have been studied extensively.^51^ Clays may be compared with MPs since both colloids break down under mechanical wear,^52^ promote biofilm formation,^53^ and are influenced by salinity via compression of the electrical double layer.^54^ However, unlike MPs, clays are largely unaffected by sunlight-driven weathering^55^ and are mostly immune to environmental thermal degradation. The high surface reactivity of clays, attributed to net negative charge and diverse functional groups, is not subject to the same stressor-induced variability of MPs. Drawing such parallels and contrasts between MPs and well-characterized environmental colloids can help elucidate their divergence in aggregation behavior, transport, and toxicity. Thus, existing knowledge can combine with the increasing research effort to provide life cycle and risk assessments on MPs as well as informing methods for their removal.

## 3 Size, Shape, and Surface Roughness of Microplastics

**Figure 2:**
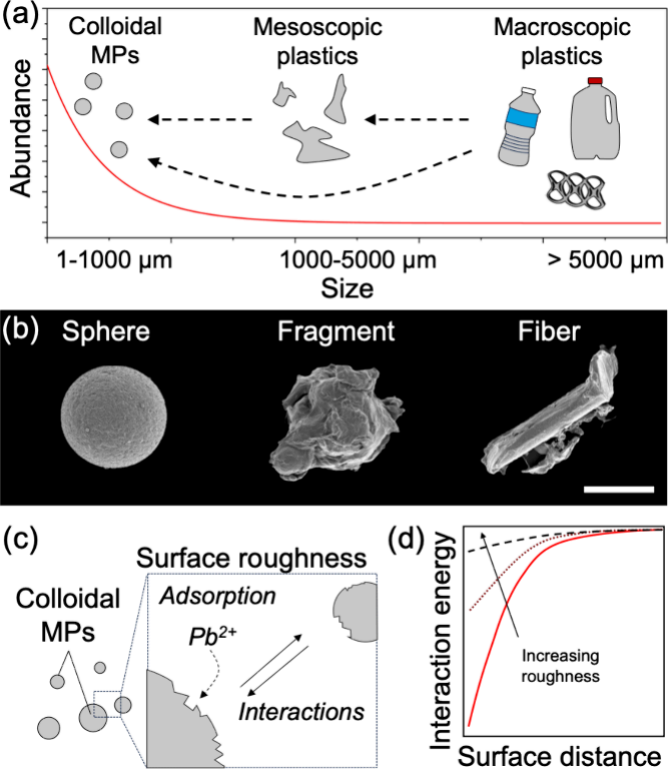
Key physical attributes of MPs. (a) Representation of a power-law distribution of the sizes of MPs as typically reported in field studies. The breakdown of plastics promotes the formation of increasingly smaller pollutants, from large millimeter-sized plastic debris to mesoscopic fragments to micro- and nanoplastics. (b) SEM micrographs of MPs with representative morphologies, i.e., spherical, fragment, and fiber. The scale bar is 50 μm. (c) Schematic representation of the nanoscale surface roughness of colloidal MPs, highlighting the role of surface roughness in adsorption and aggregation. (d) Representative extended DLVO interaction energy profiles between surfaces, displaying a decrease in attractive potential with an increase in surface roughness.

MPs are tractable as a colloidal pollutant due to their small size which makes their interfacial properties central to their behavior in the environment. However, characterizing their size distribution remains a significant technical and methodological challenge. The lack of standardized sampling, detection, and classification protocols complicates efforts to systematically review data across studies.^57^ These challenges are compounded by the heterogeneity of MPs size distributions across various environments and material compositions.^58^ Despite these issues, reports often point to power law and log-normal size distributions which reflect the tendency for progressive fragmentation of larger plastics into smaller particles^59−61^ (Figure 2a). As the size of MPs decreases down to colloidal length-scales, the role of their interfacial properties on their environmental fate becomes increasingly dominant. This is exemplified by the issues revolving around nanoplastics (NPs), defined as plastic particles smaller than 1000 nm.^62^ The inherent colloidal instability of NPs may drive them to cluster into larger aggregates, either with similar NPs forming homoaggregates, or by mixing with other particles to form heteroaggregates.^63^ This can blur the distinction between MPs and NP aggregates during detection and characterization, raising questions about the persistence of any isolated NPs in the environment. Despite these complexities, the continual fragmentation of plastics and the formation of aggregates suggest that the number density of particles increases with decreasing size. This trend is particularly concerning given the reported rise in bioavailability and ingestion of smaller MPs and NPs by marine organisms, and initial indications of potential neurotoxicity and growth rate delays among other health concerns.^64,65^

MPs occur in a wide variety of shapes, which often depend on the source of their release and the degradation pathways they undergo. For example, fiber-shaped MPs, which represent the most common form of MPs, typically arise from clothing items.^66^ Sample images of MPs with widely occurring morphologies are shown in Figure 2b. These shape variations become even more critical at the sub-10 μm scale, where shape plays a pivotal role in colloidal behavior.^67^ Specifically, the shape of MPs affects how they pack into clusters and influences their interaction landscape.^68^ Shape also significantly impacts particle transport, governing behaviors such as settling in still water^69^ and advection through porous media.^70^ While the shape of millimeter-sized MPs has been extensively studied and reported, less is known about the morphology of MPs at the micro- and nanoscale. A clearer understanding of the principles that link the morphological features of secondary MPs to the chemical composition and degradation pathways of the original macroplastic is necessary. This knowledge could enable predictions not only about the rate of MP fragmentation but also about their resulting morphology. Considering shape throughout the lifecycle of MPs, from fragmentation to aggregation and transport, can improve our ability to assess their environmental fate.

Surface roughness, much like shape, could play a key role in determining the environmental fate of MPs and is closely linked to the mechanisms that form secondary MPs.^71−73^ As macroplastics degrade, they can develop surface irregularities that increase roughness. This is normally measured using atomic force microscopy (AFM) or other optical profilometry and quantified as a root-mean-square of the feature size in the nano- or microscale.^10^ This influences the specific surface area and interfacial interactions of particles,^74^ including mm-sized MPs which may appear smooth but in fact have nanoscale asperities (Figure 2c). This increased specific surface area of rough MPs allows them to adsorb larger quantities of environmental pollutants compared to smoother particles.^75^ Additionally, surface roughness modifies the effective interactions between MPs and other natural or synthetic colloids, influencing homo- and heteroaggregation.^76^ Roughness may modulate the effective interaction involving MPs by decreasing the magnitude of short-range van der Waals attraction (Figure 2d).^74,77^ Extensions to DLVO theory are required to better interpret the effects of complex surface morphologies.^78^ In addition to the interactions, roughness affects many other interfacial phenomena that couple to determine the fate of MPs. By affecting drag forces, surface roughness influences both the vertical and horizontal transport of particles.^79,80^ Concurrently, it promotes biofilm formation,^81^ which in turn restructures the physical interface at the micro- and nanoscale.^82^ Properties such as wettability and interfacial adhesion are highly dependent on surface roughness yet are often overlooked in lifecycle assessments of MPs.

## 4 Removal of Microplastics in Wastewater Treatment Plants

Wastewater treatment plants (WWTPs) encompass various designs, with activated sludge systems being one of the most common configurations. These facilities play a significant role in removing large quantities of MPs during the treatment processes, but they are not specifically designed for MP removal. For example, WWTPs can reduce MPs in effluent streams by up to 90% as reported by Sun et al.,^24^ however the majority of these particles are retained in the sludge. While a portion of the sludge is recycled within the WWTP to sustain microbial populations for continued treatment, excess sludge is often applied as fertilizer^83^ or disposed of in landfills, effectively reintroducing MPs into the environment.^84^ Both filtering MPs from the wastewater line and preventing their escape via sludge are critical challenges. In this section, we provide a brief description of the pathway of MPs in WWTPs, and we frame the potential role of colloid-oriented solutions in addressing the key issues.

WWTP unit operations are typically divided into preliminary, primary, secondary, and tertiary treatments, all of which contribute to varying degrees of MPs removal.^24,85^ Preliminary treatment is aimed at removing bulky materials, e.g., via a combination of screening, grit removal, and flow equalization. At this stage, WWTPs may include comminution processes to break down larger materials into smaller pieces. This can inadvertently promote the fragmentation of large plastic pieces into MPs. Overall, preliminary treatment removes macroplastics via coarse screening with mesh sizes between 5 and 150 mm.^86^ The primary treatment stage involves sedimentation to remove smaller particles, including MPs, though detection limits imply that many particles remain unaccounted.^87^ Secondary treatment focuses on biological processes like activated sludge formation, which traps colloidal MPs and removes up to ∼50% of those that pass primary treatment.^86^ Tertiary treatment targets specific pollutants and uses a combination of filtration, adsorption, and chemical or biological processing to sanitize the water line before its discharge. Reported values indicate a removal by mass of up to ∼99% of MPs from the combined screening effects of all the treatments in WWTPs,^25^ yet the smallest MPs and NPs may in fact evade detection and removal.^85^

The major issue with WWTPs is the transfer of MPs from the water line to the sludge. Data reported by Li et al. on the sewage sludge from wastewater treatment plants in China indicates accumulation of MPs to an average concentration of 22.7 × 10^3^ ± 12.2 × 10^3^ particles per kg of dry sludge.^88^ However, it should be noted that many of the sampled plants in this study operate combined sewer systems, which also process stormwater, therefore the reported concentrations may reflect higher MP loads than modern separate sewer systems.^24^ Nevertheless, this poses a significant problem, as a large fraction of municipal sewage sludge is often applied to agricultural land,^89^ e.g., 80% in Ireland.^90^ Thus, as MPs pass through WWTPs, these hotspots effectively collect and concentrate MPs and subsequently reintroduce them into the environment. In addition to this crucial issue, we note that most available data on the screening efficiency of various treatment stages is given as mass fraction of MPs. Given the ambiguity in sampling and detection techniques, measurements that are agnostic of the size of the particles are likely to ignore colloidal MPs including nanoplastics. Thus, when devising and proposing new approaches for MPs removal, it is paramount to consider the fate of particles in all size regimes.

**Figure 3:**
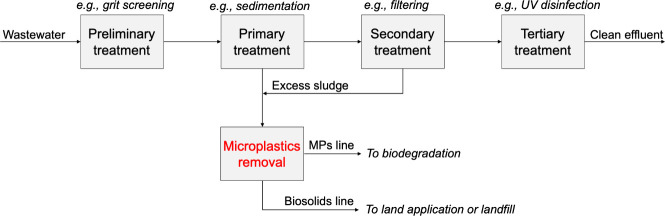
Block flow diagram of the separation of MPs in wastewater treatment plants. Influent wastewater generally undergoes preliminary, primary, secondary, and tertiary types of treatment to be discharged as clean effluent. In this process, a sludge line is formed containing biosolids, sediment, and most MPs in the wastewater. MPs should be removed from the sludge line as highlighted to minimize their re-entry into the environment via land application or landfill disposal of municipal waste.

Addressing the limitations of WWTPs requires the development of new techniques informed by principles of colloid and interfacial science. A simplified block flow diagram summarizing a general path of MPs through WWTPs and their potential removal is shown in Figure 3. Operations that revolve around density-based separation, filtration, and biochemical processing could enhance MPs removal. However, no single technology can be expected to address the current shortcomings of WWTPs. For example, in cases where coagulation and flotation^18^ are used, particles are effectively separated from the wastewater, but can allow MPs to accumulate in the sludge stream. Filters designed for molecular pollutants also face clogging issues due to MPs.^91^ This process engineering challenge calls for a new approach combining coagulation, flotation, and screening. Additionally, biochemical degradation processes can also find strong application downstream, with the use of newly discovered microbial communities that produce plastic degrading enzymes.^92^ The combination of these approaches offers a general direction for research in MPs remediation, with a focus on understanding scalable interfacial phenomena from aggregation to adsorption and engineered biofilm formation.

In addition to adapting existing WWTP processes for MPs, it is important to explore emerging approaches that leverage interfacial engineering principles. For example, Velev and co-workers recently designed environmentally benign coagulants with a dendritic nanofibrillar morphology.^18^ These biodegradable chitosan particles provide large surface area to irreversibly bind MPs via van der Waals attraction in environments with high ionic concentration. In addition, techniques that involve electromagnetic fields^93−95^ for colloidal aggregation and subsequent separation may find useful application in the removal of MPs aggregates particularly in secondary and tertiary treatment stages. Irrespective of which techniques and principles are at play, the development of new technologies for MPs removal must incorporate consideration over sustainability and integrability. The operation of wastewater management facilities requires high energy use, thus new techniques aimed at minimizing power consumption are highly desirable.^96^ Finding greener alternatives to existing toxic coagulants^97^ and energy-efficient MPs filtration systems^98^ are examples of technology that can scale up without imposing a burden on the sustainability of the plant. Concurrently, ease of integration into existing WWTP infrastructure is crucial for the practical implementation of any new process. Thus, solutions must be modular and adaptable without the need for extensive retrofitting to find faster adoption and large-scale application. Ultimately, emerging techniques for MPs removal will enhance existing WWTPs if they can be implemented while maintaining operational efficiency and reducing overall environmental impact.

## 5 Future Outlook

MPs present a multifaceted environmental challenge characterized by the colloidal length-scale of these pollutants. Their high surface-to-volume ratio makes surface forces dominate over bulk properties of MPs to drive unique interactions that impact their aggregation, pollutant adsorption, and interaction with organisms among other properties. Addressing this challenge requires a concerted effort rooted in fundamental interfacial science and engineering, with colloid scientists uniquely positioned to aid in this endeavor.^99^

**Figure 4:**
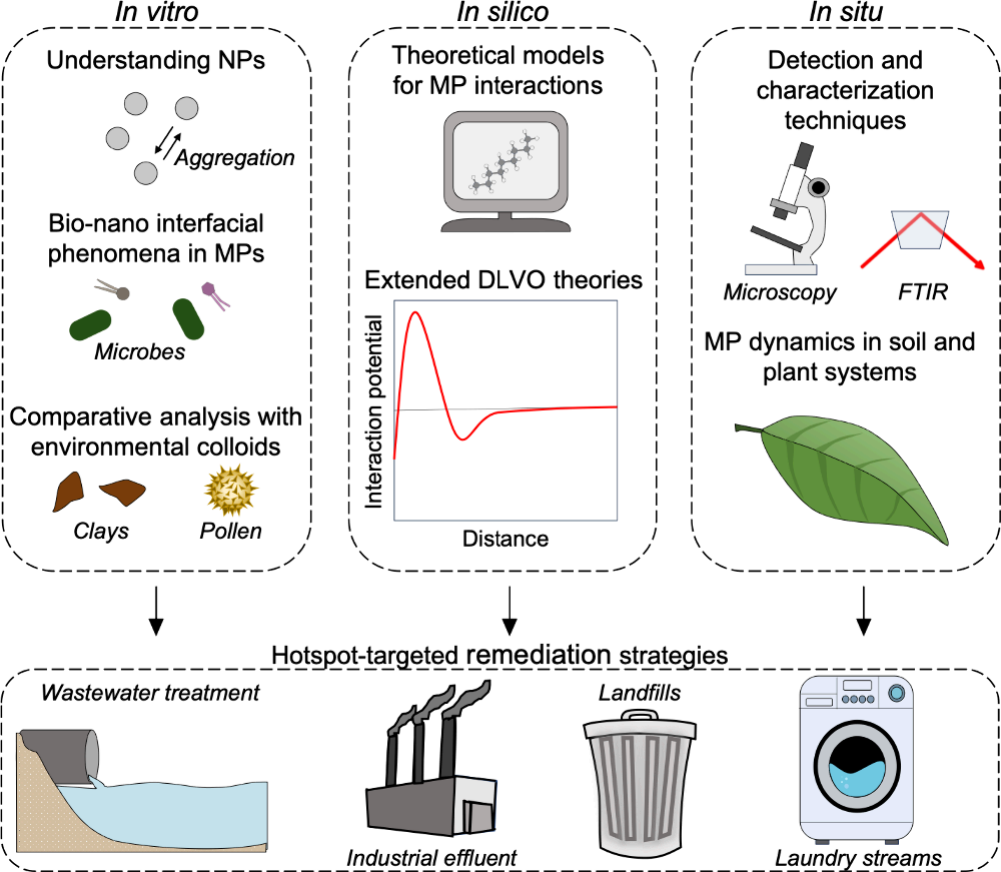
Emerging research avenues that will lead to the development of effective remediation strategies. Research areas are grouped into three categories (“*in vitro*”, “*in silico*”, and “*in situ*”), all of which will contribute to the goal of developing hotspot-targeted microplastic remediation strategies.

Looking forward, several key research avenues emerge that require careful investigation (Figure 4):

(1) Understanding nanoplastics. The persistence of isolated NPs and their transport remain among the least understood aspects of MPs pollution. Research focused on detecting NPs, understanding their aggregation mechanisms, and assessing their environmental presence is crucial.^100^
(2) Bionano interface phenomena in microplastics. MPs in biological environments interact with proteins and other biomolecules to form a corona layer. Thus, a bionano interface line of inquiry can offer novel insights on the impact of MPs on biological systems. This would require investigation of protein and biomolecule adsorption to form coronas^101^ that may attribute a “biological identity” to MPs.^102^ For example, bovine albumin^103^ and environmental DNA^104^ easily adsorb on polyethylene terephthalate due to the presence of ester groups, which facilitate increased interaction with biomolecules through hydrogen bonding. Further questions relate to the mechanisms of cellular uptake of MPs and their translocation across biological barriers.^105^
(3) Comparative analysis with environmental colloids. The release and formation of MPs in the environment invokes parallels with existing natural colloids such as clays, pollen, and organic debris, but also other potential colloidal pollutants such as engineered nanomaterials.^106^
(4) Theoretical models for microplastic interactions. Given the anticipated shortcomings of the DLVO theory for environmental MPs, it is key to develop extended models of the interaction potential. Such extended DLVO theories should incorporate more components to the total interaction energy, such as hydrophobic interactions,^107^ and account for the chemical heterogeneity and surface roughness of MPs.^78^
(5) Detection and characterization techniques. Accurate detection and characterization of MPs from the millimeter down to the nanometer scale are fundamental to advancing our understanding of their environmental impacts. It is necessary to develop sensitive analytical methods while standardizing protocols to improve the reliability of data. Microscopy and spectroscopy, as well as separation technologies will be instrumental in monitoring MPs pollution and evaluating the effectiveness of remediation efforts.^108−111^ A recent issue of *ACS ES&T* highlights notable advancements, including the use of hyperspectral imaging for rapid MPs type and size characterization, and the integration of machine learning for analyzing spectroscopic data on MPs.^15^
(6) Microplastic dynamics in soil and plant systems. MPs are ubiquitous in soil matrices globally,^110^ yet little is known about the effect of confinement imposed by the soil grains on the particles. Concurrently, the potential role of MPs on soil microbiome, nutrient cycling, and plant health is vital for assessing environmental risks.^112,113^
(7) Hotspot-targeted remediation strategies. Beyond WWTPs, several other hotspots exist which present unique opportunities for colloid science-driven solutions. Domestic laundry processes release significant amounts of fiber-shaped MPs from synthetic textiles that necessitate dedicated filtration and separation technology.^114^ Similarly, industrial effluents are major sources of MPs that require tailored approaches for MPs removal.

Addressing the environmental challenges posed by MPs requires an interdisciplinary approach that harnesses expertise in chemical engineering, physics, biology, environmental science, process engineering, and policy. We believe that a nuanced knowledge of colloid and interfacial science forms the central component in this collective endeavor.

## References

[ref1] Hale R. C.; Seeley M. E.; La Guardia M. J.; Mai L.; Zeng E. Y. A Global Perspective on Microplastics. J. Geophys Res. Oceans 2020, 125 (1), e2018JC014719 10.1029/2018JC014719.

[ref2] Wang T.; Li B.; Zou X.; Wang Y.; Li Y.; Xu Y.; Mao L.; Zhang C.; Yu W. Emission of Primary Microplastics in Mainland China: Invisible but Not Negligible. Water Res. 2019, 162, 214–224. 10.1016/j.watres.2019.06.042. 31276985

[ref3] Kukkola A.; Chetwynd A. J.; Krause S.; Lynch I. Beyond Microbeads: Examining the Role of Cosmetics in Microplastic Pollution and Spotlighting Unanswered Questions. J. Hazard Mater. 2024, 476, 135053 10.1016/j.jhazmat.2024.135053. 38976961

[ref4] Singh A.; Mishra B. K. Microbeads in Personal Care Products: An Overlooked Environmental Concern. J. Clean Prod 2023, 427, 139082 10.1016/j.jclepro.2023.139082.

[ref5] Xanthos D.; Walker T. R. International Policies to Reduce Plastic Marine Pollution from Single-Use Plastics (Plastic Bags and Microbeads): A Review. Mar. Pollut. Bull. 2017, 118 (1), 17–26. 10.1016/j.marpolbul.2017.02.048. 28238328

[ref6] Al Harraq A.; Bharti B. Microplastics through the Lens of Colloid Science. ACS Environmental Au 2022, 2 (1), 3–10. 10.1021/acsenvironau.1c00016. 37101760 PMC10125150

[ref7] Zhao X.; You F. Life Cycle Assessment of Microplastics Reveals Their Greater Environmental Hazards than Mismanaged Polymer Waste Losses. Environ. Sci. Technol. 2022, 56 (16), 11780–11797. 10.1021/acs.est.2c01549. 35920730

[ref8] Pete A. J.; Brahana P. J.; Bello M.; Benton M. G.; Bharti B. Biofilm Formation Influences the Wettability and Settling of Microplastics. Environ. Sci. Technol. Lett. 2023, 10 (2), 159–164. 10.1021/acs.estlett.2c00728.

[ref9] Al Harraq A.; Brahana P. J.; Arcemont O.; Zhang D.; Valsaraj K. T.; Bharti B. Effects of Weathering on Microplastic Dispersibility and Pollutant Uptake Capacity. ACS Environmental Au 2022, 2 (6), 549–555. 10.1021/acsenvironau.2c00036. 36411868 PMC9673469

[ref10] Brahana P.; Zhang M.; Nakouzi E.; Bharti B. Weathering Influences the Ice Nucleation Activity of Microplastics. Nat. Commun. 2024, 15 (1), 9579 10.1038/s41467-024-53987-8. 39505887 PMC11542094

[ref11] Brahana P. J.; Al Harraq A.; Saab L. E.; Roberg R.; Valsaraj K. T.; Bharti B. Uptake and Release of Perfluoroalkyl Carboxylic Acids (PFCAs) from Macro and Microplastics. Environ. Sci. Process Impacts 2023, 25 (9), 1519–1531. 10.1039/D3EM00209H. 37602395

[ref12] Wang L.; Yang H.; Guo M.; Wang Z.; Zheng X. Adsorption of Antibiotics on Different Microplastics (MPs): Behavior and Mechanism. Science of The Total Environment 2023, 863, 161022 10.1016/j.scitotenv.2022.161022. 36549518

[ref13] Mesquita Pinheiro L.; Ivar do Sul J. A.; Costa M. Uptake and Ingestion Are the Main Pathways for Microplastics to Enter Marine Benthos: A Review. Food Webs 2020, 24, e00150 10.1016/j.fooweb.2020.e00150.

[ref14] Jovanović B. Ingestion of Microplastics by Fish and Its Potential Consequences from a Physical Perspective. Integr Environ. Assess Manag 2017, 13 (3), 510–515. 10.1002/ieam.1913. 28440941

[ref15] Mitrano D. M.; Diamond M. L.; Kim J.-H.; Tam K. C.; Yang M.; Wang Z. Balancing New Approaches and Harmonized Techniques in Nano- and Microplastics Research. Environ. Sci. Technol. 2023, 57 (24), 8841–8844. 10.1021/acs.est.3c04120. 37283499

[ref16] Wang X.; Li Y.; Kroll A.; Mitrano D. M. Differentiating Microplastics from Natural Particles in Aqueous Suspensions Using Flow Cytometry with Machine Learning. Environ. Sci. Technol. 2024, 58 (23), 10240–10251. 10.1021/acs.est.4c00304. 38803057

[ref17] Sarcletti M.; Park H.; Wirth J.; Englisch S.; Eigen A.; Drobek D.; Vivod D.; Friedrich B.; Tietze R.; Alexiou C.; Zahn D.; Apeleo Zubiri B.; Spiecker E.; Halik M. The Remediation of Nano-/Microplastics from Water. Mater. Today 2021, 48, 38–46. 10.1016/j.mattod.2021.02.020.

[ref18] Bang R. S.; Verster L.; Hong H.; Pal L.; Velev O. D. Colloidal Engineering of Microplastic Capture with Biodegradable Soft Dendritic “Microcleaners.. Langmuir 2024, 40 (11), 5923–5933. 10.1021/acs.langmuir.3c03869. 38428025

[ref19] Padervand M.; Lichtfouse E.; Robert D.; Wang C. Removal of Microplastics from the Environment. A Review. Environ. Chem. Lett. 2020, 18 (3), 807–828. 10.1007/s10311-020-00983-1.

[ref20] Calero M.; Godoy V.; Quesada L.; Martín-Lara M. Á. Green Strategies for Microplastics Reduction. Curr. Opin Green Sustain Chem. 2021, 28, 100442 10.1016/j.cogsc.2020.100442.

[ref21] Hunt C. F.; Lin W. H.; Voulvoulis N. Evaluating Alternatives to Plastic Microbeads in Cosmetics. Nat. Sustain 2021, 4 (4), 366–372. 10.1038/s41893-020-00651-w.

[ref22] Sun C.; Wang Z.; Chen L.; Li F. Fabrication of Robust and Compressive Chitin and Graphene Oxide Sponges for Removal of Microplastics with Different Functional Groups. Chemical Engineering Journal 2020, 393, 124796 10.1016/j.cej.2020.124796.

[ref23] Yogarathinam L. T.; Usman J.; Othman M. H. D.; Ismail A. F.; Goh P. S.; Gangasalam A.; Adam M. R. Low-Cost Silica Based Ceramic Supported Thin Film Composite Hollow Fiber Membrane from Guinea Corn Husk Ash for Efficient Removal of Microplastic from Aqueous Solution. J. Hazard Mater. 2022, 424, 127298 10.1016/j.jhazmat.2021.127298. 34571470

[ref24] Sun J.; Dai X.; Wang Q.; van Loosdrecht M. C. M.; Ni B.-J. Microplastics in Wastewater Treatment Plants: Detection, Occurrence and Removal. Water Res. 2019, 152, 21–37. 10.1016/j.watres.2018.12.050. 30660095

[ref25] Xu Z.; Bai X.; Ye Z. Removal and Generation of Microplastics in Wastewater Treatment Plants: A Review. J. Clean Prod 2021, 291, 125982 10.1016/j.jclepro.2021.125982.

[ref26] Hou L.; Kumar D.; Yoo C. G.; Gitsov I.; Majumder E. L.-W. Conversion and Removal Strategies for Microplastics in Wastewater Treatment Plants and Landfills. Chemical Engineering Journal 2021, 406, 126715 10.1016/j.cej.2020.126715.

[ref27] Rolsky C.; Kelkar V.; Driver E.; Halden R. U. Municipal Sewage Sludge as a Source of Microplastics in the Environment. Curr. Opin Environ. Sci. Health 2020, 14, 16–22. 10.1016/j.coesh.2019.12.001.

[ref28] Costa J. P. da; Avellan A.; Mouneyrac C.; Duarte A.; Rocha-Santos T. Plastic Additives and Microplastics as Emerging Contaminants: Mechanisms and Analytical Assessment. TrAC Trends in Analytical Chemistry 2023, 158, 116898 10.1016/j.trac.2022.116898.

[ref29] Sun A.; Wang W.-X. Photodegradation of Microplastics by ZnO Nanoparticles with Resulting Cellular and Subcellular Responses. Environ. Sci. Technol. 2023, 57 (21), 8118–8129. 10.1021/acs.est.3c01307. 37192337

[ref30] Al-Darraji A.; Oluwoye I.; Lagat C.; Tanaka S.; Barifcani A. Erosion of Rigid Plastics in Turbid (Sandy) Water: Quantitative Assessment for Marine Environments and Formation of Microplastics. Environ. Sci. Process Impacts 2024, 26 (10), 1847–1858. 10.1039/D4EM00122B. 39221511

[ref31] Bullard J. E.; Zhou Z.; Davis S.; Fowler S. Breakdown and Modification of Microplastic Beads by Aeolian Abrasion. Environ. Sci. Technol. 2023, 57 (1), 76–84. 10.1021/acs.est.2c05396. 36519925 PMC9835823

[ref32] Wu X.; Pan J.; Li M.; Li Y.; Bartlam M.; Wang Y. Selective Enrichment of Bacterial Pathogens by Microplastic Biofilm. Water Res. 2019, 165, 114979 10.1016/j.watres.2019.114979. 31445309

[ref33] Yu Y.; Kumar M.; Bolan S.; Padhye L. P.; Bolan N.; Li S.; Wang L.; Hou D.; Li Y. Various Additive Release from Microplastics and Their Toxicity in Aquatic Environments. Environ. Pollut. 2024, 343, 123219 10.1016/j.envpol.2023.123219. 38154772

[ref34] Yurtsever M. Glitters as a Source of Primary Microplastics: An Approach to Environmental Responsibility and Ethics. J. Agric Environ. Ethics 2019, 32 (3), 459–478. 10.1007/s10806-019-09785-0.

[ref35] Piccardo M.; Provenza F.; Anselmi S.; Renzi M. Ecotoxicological Assessment of “Glitter” Leachates in Aquatic Ecosystems: An Integrated Approach. Toxics 2022, 10 (11), 677 10.3390/toxics10110677. 36355968 PMC9697108

[ref36] Golmohammadi M.; Fatemeh Musavi S.; Habibi M.; Maleki R.; Golgoli M.; Zargar M.; Dumée L. F.; Baroutian S.; Razmjou A. Molecular Mechanisms of Microplastics Degradation: A Review. Sep Purif Technol. 2023, 309, 122906 10.1016/j.seppur.2022.122906.

[ref37] Khaled A.; Rivaton A.; Richard C.; Jaber F.; Sleiman M. Phototransformation of Plastic Containing Brominated Flame Retardants: Enhanced Fragmentation and Release of Photoproducts to Water and Air. Environ. Sci. Technol. 2018, 52 (19), 11123–11131. 10.1021/acs.est.8b03172. 30169020

[ref38] Lu S.; Zhu K.; Song W.; Song G.; Chen D.; Hayat T.; Alharbi N. S.; Chen C.; Sun Y. Impact of Water Chemistry on Surface Charge and Aggregation of Polystyrene Microspheres Suspensions. Science of The Total Environment 2018, 630, 951–959. 10.1016/j.scitotenv.2018.02.296. 29499550

[ref39] Liu F.; Liu G.; Zhu Z.; Wang S.; Zhao F. Interactions between Microplastics and Phthalate Esters as Affected by Microplastics Characteristics and Solution Chemistry. Chemosphere 2019, 214, 688–694. 10.1016/j.chemosphere.2018.09.174. 30292051

[ref40] Tan M.; Liu L.; Zhang M.; Liu Y.; Li C. Effects of Solution Chemistry and Humic Acid on the Transport of Polystyrene Microplastics in Manganese Oxides Coated Sand. J. Hazard Mater. 2021, 413, 125410 10.1016/j.jhazmat.2021.125410. 33611036

[ref41] Parrish K.; Fahrenfeld N. L. Microplastic Biofilm in Fresh- and Wastewater as a Function of Microparticle Type and Size Class. Environ. Sci.: Water Res. Technol. 2019, 5 (3), 495–505. 10.1039/C8EW00712H.

[ref42] Wang J.; Lu J.; Zhang Y.; Wu J.; Luo Y. Unique Bacterial Community of the Biofilm on Microplastics in Coastal Water. Bull. Environ. Contam. Toxicol. 2021, 107 (4), 597–601. 10.1007/s00128-020-02875-0. 32417953

[ref43] Lee S. W.; Carnicelli J.; Getya D.; Gitsov I.; Phillips K. S.; Ren D. Biofilm Removal by Reversible Shape Recovery of the Substrate. ACS Appl. Mater. Interfaces 2021, 13 (15), 17174–17182. 10.1021/acsami.0c20697. 33822590 PMC8153534

[ref44] Li Y.; Wang X.; Fu W.; Xia X.; Liu C.; Min J.; Zhang W.; Crittenden J. C. Interactions between Nano/Micro Plastics and Suspended Sediment in Water: Implications on Aggregation and Settling. Water Res. 2019, 161, 486–495. 10.1016/j.watres.2019.06.018. 31229729

[ref45] Liu L.; Song J.; Zhang M.; Jiang W. Aggregation and Deposition Kinetics of Polystyrene Microplastics and Nanoplastics in Aquatic Environment. Bull. Environ. Contam. Toxicol. 2021, 107 (4), 741–747. 10.1007/s00128-021-03239-y. 33914100

[ref46] Bhattacharjee S.; Chen J. Y.; Elimelech M. DLVO Interaction Energy between Spheroidal Particles and a Flat Surface. Colloids Surf. A Physicochem Eng. Asp 2000, 165 (1), 143–156. 10.1016/S0927-7757(99)00448-3.

[ref47] Guo Y.; Tang N.; Guo J.; Lu L.; Li N.; Hu T.; Zhu Z.; Gao X.; Li X.; Jiang L.; Liang J. The Aggregation of Natural Inorganic Colloids in Aqueous Environment: A Review. Chemosphere 2023, 310, 136805 10.1016/j.chemosphere.2022.136805. 36223821

[ref48] Zhu L.; Xie C.; Chen L.; Dai X.; Zhou Y.; Pan H.; Tian K. Transport of Microplastics in the Body and Interaction with Biological Barriers, and Controlling of Microplastics Pollution. Ecotoxicol Environ. Saf 2023, 255, 114818 10.1016/j.ecoenv.2023.114818. 36958263

[ref49] Nel A. E.; Mädler L.; Velegol D.; Xia T.; Hoek E. M. V; Somasundaran P.; Klaessig F.; Castranova V.; Thompson M. Understanding Biophysicochemical Interactions at the Nano-Bio Interface. Nat. Mater. 2009, 8 (7), 543–557. 10.1038/nmat2442. 19525947

[ref50] Schvartz M.; Saudrais F.; Devineau S.; Chédin S.; Jamme F.; Leroy J.; Rakotozandriny K.; Taché O.; Brotons G.; Pin S.; Boulard Y.; Renault J.-P. Role of the Protein Corona in the Colloidal Behavior of Microplastics. Langmuir 2023, 39 (12), 4291–4303. 10.1021/acs.langmuir.2c03237. 36930733

[ref51] Sposito G.; Skipper N. T.; Sutton R.; Park S.; Soper A. K.; Greathouse J. A. Surface Geochemistry of the Clay Minerals. Proc. Natl. Acad. Sci. U. S. A. 1999, 96 (7), 3358–3364. 10.1073/pnas.96.7.3358. 10097044 PMC34275

[ref52] Attou F.; Bruand A.; Le Bissonnais Y. Effect of Clay Content and Silt—Clay Fabric on Stability of Artificial Aggregates. Eur. J. Soil Sci. 1998, 49 (4), 569–577. 10.1046/j.1365-2389.1998.4940569.x.

[ref53] Ma W.; Peng D.; Walker S. L.; Cao B.; Gao C.-H.; Huang Q.; Cai P. Bacillus Subtilis Biofilm Development in the Presence of Soil Clay Minerals and Iron Oxides. NPJ. Biofilms Microbiomes 2017, 3 (1), 4 10.1038/s41522-017-0013-6. 28649405 PMC5445608

[ref54] Xu C.-Y.; Yu Z.-H.; Li H. The Coupling Effects of Electric Field and Clay Mineralogy on Clay Aggregate Stability. J. Soils Sediments 2015, 15 (5), 1159–1168. 10.1007/s11368-015-1063-0.

[ref55] Phua S. L.; Yang L.; Toh C. L.; Guoqiang D.; Lau S. K.; Dasari A.; Lu X. Simultaneous Enhancements of UV Resistance and Mechanical Properties of Polypropylene by Incorporation of Dopamine-Modified Clay. ACS Appl. Mater. Interfaces 2013, 5 (4), 1302–1309. 10.1021/am3024405. 23360646

[ref56] Najjar K.; Bridge C. M. SEM-EDS Analysis and Characterization of Glitter and Shimmer Cosmetic Particles. Forensic Sci. Int. 2020, 317, 110527 10.1016/j.forsciint.2020.110527. 33065447

[ref57] Caputo F.; Vogel R.; Savage J.; Vella G.; Law A.; Della Camera G.; Hannon G.; Peacock B.; Mehn D.; Ponti J.; Geiss O.; Aubert D.; Prina-Mello A.; Calzolai L. Measuring Particle Size Distribution and Mass Concentration of Nanoplastics and Microplastics: Addressing Some Analytical Challenges in the Sub-Micron Size Range. J. Colloid Interface Sci. 2021, 588, 401–417. 10.1016/j.jcis.2020.12.039. 33422789

[ref58] Kameda Y.; Yamada N.; Fujita E. Source- and Polymer-Specific Size Distributions of Fine Microplastics in Surface Water in an Urban River. Environ. Pollut. 2021, 284, 117516 10.1016/j.envpol.2021.117516. 34261221

[ref59] Kooi M.; Koelmans A. A. Simplifying Microplastic via Continuous Probability Distributions for Size, Shape, and Density. Environ. Sci. Technol. Lett. 2019, 6 (9), 551–557. 10.1021/acs.estlett.9b00379.

[ref60] Kooi M.; Primpke S.; Mintenig S. M.; Lorenz C.; Gerdts G.; Koelmans A. A. Characterizing the Multidimensionality of Microplastics across Environmental Compartments. Water Res. 2021, 202, 117429 10.1016/j.watres.2021.117429. 34304075

[ref61] Jian M.; Zhang Y.; Yang W.; Zhou L.; Liu S.; Xu E. G. Occurrence and Distribution of Microplastics in China’s Largest Freshwater Lake System. Chemosphere 2020, 261, 128186 10.1016/j.chemosphere.2020.128186. 33113661

[ref62] Hartmann N. B.; Hüffer T.; Thompson R. C.; Hassellöv M.; Verschoor A.; Daugaard A. E.; Rist S.; Karlsson T.; Brennholt N.; Cole M.; Herrling M. P.; Hess M. C.; Ivleva N. P.; Lusher A. L.; Wagner M. Are We Speaking the Same Language? Recommendations for a Definition and Categorization Framework for Plastic Debris. Environ. Sci. Technol. 2019, 53 (3), 1039–1047. 10.1021/acs.est.8b05297. 30608663

[ref63] Piccardo M.; Renzi M.; Terlizzi A. Nanoplastics in the Oceans: Theory, Experimental Evidence and Real World. Mar. Pollut. Bull. 2020, 157, 111317 10.1016/j.marpolbul.2020.111317. 32658682

[ref64] Lehtiniemi M.; Hartikainen S.; Näkki P.; Engström-Öst J.; Koistinen A.; Setälä O. Size Matters More than Shape: Ingestion of Primary and Secondary Microplastics by Small Predators. Food Webs 2018, 17, e00097 10.1016/j.fooweb.2018.e00097.

[ref65] de Sá L. C.; Oliveira M.; Ribeiro F.; Rocha T. L.; Futter M. N. Studies of the Effects of Microplastics on Aquatic Organisms: What Do We Know and Where Should We Focus Our Efforts in the Future?. Science of The Total Environment 2018, 645, 1029–1039. 10.1016/j.scitotenv.2018.07.207. 30248828

[ref66] Chen Q.; Yang Y.; Qi H.; Su L.; Zuo C.; Shen X.; Chu W.; Li F.; Shi H. Rapid Mass Conversion for Environmental Microplastics of Diverse Shapes. Environ. Sci. Technol. 2024, 58 (24), 10776–10785. 10.1021/acs.est.4c01031. 38838101

[ref67] Liu F.; Rasmussen L. A.; Klemmensen N. D. R.; Zhao G.; Nielsen R.; Vianello A.; Rist S.; Vollertsen J. Shapes of Hyperspectral Imaged Microplastics. Environ. Sci. Technol. 2023, 57 (33), 12431–12441. 10.1021/acs.est.3c03517. 37561646 PMC10448723

[ref68] Glotzer S. C.; Solomon M. J. Anisotropy of Building Blocks and Their Assembly into Complex Structures. Nat. Mater. 2007, 6 (8), 557–562. 10.1038/nmat1949. 17667968

[ref69] Senis D.; Gorre-Talini L.; Allain C. Systematic Study of the Settling Kinetics in an Aggregating Colloidal Suspension. Eur. Phys. J. E 2001, 4 (1), 59–68. 10.1007/PL00013683.

[ref70] Seymour M. B.; Chen G.; Su C.; Li Y. Transport and Retention of Colloids in Porous Media: Does Shape Really Matter?. Environ. Sci. Technol. 2013, 47 (15), 8391–8398. 10.1021/es4016124. 23822811

[ref71] Song Y. K.; Hong S. H.; Jang M.; Han G. M.; Jung S. W.; Shim W. J. Combined Effects of UV Exposure Duration and Mechanical Abrasion on Microplastic Fragmentation by Polymer Type. Environ. Sci. Technol. 2017, 51 (8), 4368–4376. 10.1021/acs.est.6b06155. 28249388

[ref72] Sun J.; Zheng H.; Xiang H.; Fan J.; Jiang H. The Surface Degradation and Release of Microplastics from Plastic Films Studied by UV Radiation and Mechanical Abrasion. Science of The Total Environment 2022, 838, 156369 10.1016/j.scitotenv.2022.156369. 35654205

[ref73] Fotopoulou K. N.; Karapanagioti H. K. Surface Properties of Beached Plastics. Environmental Science and Pollution Research 2015, 22 (14), 11022–11032. 10.1007/s11356-015-4332-y. 25787219

[ref74] Guillot K. A.; Brahana P. J.; Al Harraq A.; Ogbonna N. D.; Lombardo N. S.; Lawrence J.; An Y.; Benton M. G.; Bharti B. Selective Vapor Condensation for the Synthesis and Assembly of Spherical Colloids with a Precise Rough Patch. JACS Au 2024, 4 (3), 1107–1117. 10.1021/jacsau.3c00812. 38559733 PMC10976603

[ref75] Wang Z.; Chen M.; Zhang L.; Wang K.; Yu X.; Zheng Z.; Zheng R. Sorption Behaviors of Phenanthrene on the Microplastics Identified in a Mariculture Farm in Xiangshan Bay, Southeastern China. Science of The Total Environment 2018, 628–629, 1617–1626. 10.1016/j.scitotenv.2018.02.146. 30045578

[ref76] Mukherjee F.; Shi A.; Wang X.; You F.; Abbott N. L. Liquid Crystals as Multifunctional Interfaces for Trapping and Characterizing Colloidal Microplastics. Small 2023, 19 (23), 2207802 10.1002/smll.202207802. 36892170

[ref77] Rajupet S.; Riet A. A.; Chen Q.; Sow M.; Lacks D. J. Relative Importance of Electrostatic and van Der Waals Forces in Particle Adhesion to Rough Conducting Surfaces. Phys. Rev. E 2021, 103 (4), 42906 10.1103/PhysRevE.103.042906. 34005883

[ref78] Hoek E. M. V; Agarwal G. K. Extended DLVO Interactions between Spherical Particles and Rough Surfaces. J. Colloid Interface Sci. 2006, 298 (1), 50–58. 10.1016/j.jcis.2005.12.031. 16469325

[ref79] Rasmuson A.; VanNess K.; Ron C. A.; Johnson W. P. Hydrodynamic versus Surface Interaction Impacts of Roughness in Closing the Gap between Favorable and Unfavorable Colloid Transport Conditions. Environ. Sci. Technol. 2019, 53 (5), 2450–2459. 10.1021/acs.est.8b06162. 30762346

[ref80] Li J.; Shan E.; Zhao J.; Teng J.; Wang Q. The Factors Influencing the Vertical Transport of Microplastics in Marine Environment: A Review. Science of The Total Environment 2023, 870, 161893 10.1016/j.scitotenv.2023.161893. 36731545

[ref81] Ammar Y.; Swailes D.; Bridgens B.; Chen J. Influence of Surface Roughness on the Initial Formation of Biofilm. Surf. Coat. Technol. 2015, 284, 410–416. 10.1016/j.surfcoat.2015.07.062.

[ref82] Meng D.; Li Y. Assessing the Settling Velocity of Biofilm-Encrusted Microplastics: Accounting for Biofilms as an Equivalent to Surface Roughness. Environ. Sci. Technol. 2024, 58 (2), 1329–1337. 10.1021/acs.est.3c07147. 38163930

[ref83] Yang J.; Li L.; Li R.; Xu L.; Shen Y.; Li S.; Tu C.; Wu L.; Christie P.; Luo Y. Microplastics in an Agricultural Soil Following Repeated Application of Three Types of Sewage Sludge: A Field Study. Environ. Pollut. 2021, 289, 117943 10.1016/j.envpol.2021.117943. 34426179

[ref84] Woodward J.; Li J.; Rothwell J.; Hurley R. Acute Riverine Microplastic Contamination Due to Avoidable Releases of Untreated Wastewater. Nat. Sustain 2021, 4 (9), 793–802. 10.1038/s41893-021-00718-2.

[ref85] Ziajahromi S.; Neale P. A.; Rintoul L.; Leusch F. D. L. Wastewater Treatment Plants as a Pathway for Microplastics: Development of a New Approach to Sample Wastewater-Based Microplastics. Water Res. 2017, 112, 93–99. 10.1016/j.watres.2017.01.042. 28160700

[ref86] Iyare P. U.; Ouki S. K.; Bond T. Microplastics Removal in Wastewater Treatment Plants: A Critical Review. Environ. Sci.: Water Res. Technol. 2020, 6 (10), 2664–2675. 10.1039/D0EW00397B.

[ref87] Elkhatib D.; Oyanedel-Craver V. A Critical Review of Extraction and Identification Methods of Microplastics in Wastewater and Drinking Water. Environ. Sci. Technol. 2020, 54 (12), 7037–7049. 10.1021/acs.est.9b06672. 32432459

[ref88] Li X.; Chen L.; Mei Q.; Dong B.; Dai X.; Ding G.; Zeng E. Y. Microplastics in Sewage Sludge from the Wastewater Treatment Plants in China. Water Res. 2018, 142, 75–85. 10.1016/j.watres.2018.05.034. 29859394

[ref89] Kelessidis A.; Stasinakis A. S. Comparative Study of the Methods Used for Treatment and Final Disposal of Sewage Sludge in European Countries. Waste Management 2012, 32 (6), 1186–1195. 10.1016/j.wasman.2012.01.012. 22336390

[ref90] Hudcová H.; Vymazal J.; Rozkošný M. Present Restrictions of Sewage Sludge Application in Agriculture within the European Union. Soil and Water Research 2019, 14 (2), 104–120. 10.17221/36/2018-SWR.

[ref91] Hube S.; Eskafi M.; Hrafnkelsdóttir K. F.; Bjarnadóttir B.; Bjarnadóttir M. Á.; Axelsdóttir S.; Wu B. Direct Membrane Filtration for Wastewater Treatment and Resource Recovery: A Review. Science of The Total Environment 2020, 710, 136375 10.1016/j.scitotenv.2019.136375. 31923693

[ref92] Zurier H. S.; Goddard J. M. Biodegradation of Microplastics in Food and Agriculture. Curr. Opin Food Sci. 2021, 37, 37–44. 10.1016/j.cofs.2020.09.001.

[ref93] Harraq A. Al; Choudhury B. D.; Bharti B. Field-Induced Assembly and Propulsion of Colloids. Langmuir 2022, 38 (10), 3001–3016. 10.1021/acs.langmuir.1c02581. 35238204 PMC8928473

[ref94] Shen M.; Zhang Y.; Almatrafi E.; Hu T.; Zhou C.; Song B.; Zeng Z.; Zeng G. Efficient Removal of Microplastics from Wastewater by an Electrocoagulation Process. Chemical Engineering Journal 2022, 428, 131161 10.1016/j.cej.2021.131161.

[ref95] Shi C.; Zhang S.; Zhao J.; Ma J.; Wu H.; Sun H.; Cheng S. Experimental Study on Removal of Microplastics from Aqueous Solution by Magnetic Force Effect on the Magnetic Sepiolite. Sep Purif Technol. 2022, 288, 120564 10.1016/j.seppur.2022.120564.

[ref96] Ghimire U.; Sarpong G.; Gude V. G. Transitioning Wastewater Treatment Plants toward Circular Economy and Energy Sustainability. ACS Omega 2021, 6 (18), 11794–11803. 10.1021/acsomega.0c05827. 34056333 PMC8154022

[ref97] Ang W. L.; Mohammad A. W. State of the Art and Sustainability of Natural Coagulants in Water and Wastewater Treatment. J. Clean Prod 2020, 262, 121267 10.1016/j.jclepro.2020.121267.

[ref98] Arkhangelsky E.; Levitsky I.; Gitis V. Considering Energy Efficiency in Filtration of Engineering Nanoparticles. Water Supply 2017, 17 (5), 1212–1218. 10.2166/ws.2017.023.

[ref99] Bang R. S.; Bergman M.; Li T.; Mukherjee F.; Alshehri A. S.; Abbott N. L.; Crook N. C.; Velev O. D.; Hall C. K.; You F. An Integrated Chemical Engineering Approach to Understanding Microplastics. AIChE J. 2023, 69 (4), e18020 10.1002/aic.18020.

[ref100] Pradel A.; Catrouillet C.; Gigault J. The Environmental Fate of Nanoplastics: What We Know and What We Need to Know about Aggregation. NanoImpact 2023, 29, 100453 10.1016/j.impact.2023.100453. 36708989

[ref101] Luo H.; Du Q.; Zhong Z.; Xu Y.; Peng J. Protein-Coated Microplastics Corona Complex: An Underestimated Risk of Microplastics. Science of The Total Environment 2022, 851, 157948 10.1016/j.scitotenv.2022.157948. 35963400

[ref102] Vasti C.; Bonnet L. V.; Galiano M. R.; Rojas R.; Giacomelli C. E. Relevance of Protein-Protein Interactions on the Biological Identity of Nanoparticles. Colloids Surf. B Biointerfaces 2018, 166, 330–338. 10.1016/j.colsurfb.2018.03.032. 29609156

[ref103] Beyer D.; Knoll W.; Ringsdorf H.; Wang J.-H.; Timmons R. B.; Sluka P. Reduced Protein Adsorption on Plastics via Direct Plasma Deposition of Triethylene Glycol Monoallyl Ether. J. Biomed Mater. Res. 1997, 36 (2), 181–189. 10.1002/(SICI)1097-4636(199708)36:2<181::AID-JBM6>3.0.CO;2-G. 9261679

[ref104] Müller N. D.; Kirtane A.; Schefer R. B.; Mitrano D. M. EDNA Adsorption onto Microplastics: Impacts of Water Chemistry and Polymer Physiochemical Properties. Environ. Sci. Technol. 2024, 58 (17), 7588–7599. 10.1021/acs.est.3c10825. 38624040

[ref105] Zhao X.; Sun J.; Zhou L.; Teng M.; Zhao L.; Li Y.; Wu F. Defining the Size Ranges of Polystyrene Nanoplastics According to Their Ability to Cross Biological Barriers. Environ. Sci. Nano 2023, 10 (10), 2634–2645. 10.1039/D3EN00491K.

[ref106] Guinée J. B.; Heijungs R.; Vijver M. G.; Peijnenburg W. J. G. M. Setting the Stage for Debating the Roles of Risk Assessment and Life-Cycle Assessment of Engineered Nanomaterials. Nat. Nanotechnol 2017, 12 (8), 727–733. 10.1038/nnano.2017.135. 28775351

[ref107] Ivanic F. M.; Guggenberger G.; Woche S. K.; Bachmann J.; Hoppe M.; Carstens J. F. Soil Organic Matter Facilitates the Transport of Microplastic by Reducing Surface Hydrophobicity. Colloids Surf. A Physicochem Eng. Asp 2023, 676, 132255 10.1016/j.colsurfa.2023.132255.

[ref108] Primpke S.; Godejohann M.; Gerdts G. Rapid Identification and Quantification of Microplastics in the Environment by Quantum Cascade Laser-Based Hyperspectral Infrared Chemical Imaging. Environ. Sci. Technol. 2020, 54 (24), 15893–15903. 10.1021/acs.est.0c05722. 33233891

[ref109] Hildebrandt L.; Mitrano D. M.; Zimmermann T.; Pröfrock D. A Nanoplastic Sampling and Enrichment Approach by Continuous Flow Centrifugation. Front. Environ. Sci. 2020, 8, 89 10.3389/fenvs.2020.00089.

[ref110] Ribeiro-Claro P.; Nolasco M. M.; Araújo C. Characterization of Microplastics by Raman Spectroscopy. Compr. Anal. Chem. 2017, 75, 119–151. 10.1016/bs.coac.2016.10.001.

[ref111] Möller J. N.; Löder M. G. J.; Laforsch C. Finding Microplastics in Soils: A Review of Analytical Methods. Environ. Sci. Technol. 2020, 54 (4), 2078–2090. 10.1021/acs.est.9b04618. 31999440

[ref112] Rillig M. C.; Kim S. W.; Zhu Y.-G. The Soil Plastisphere. Nat. Rev. Microbiol 2024, 22 (2), 64–74. 10.1038/s41579-023-00967-2. 37697003 PMC7615554

[ref113] Liu Y.; Xu F.; Ding L.; Zhang G.; Bai B.; Han Y.; Xiao L.; Song Y.; Li Y.; Wan S.; Li G. Microplastics Reduce Nitrogen Uptake in Peanut Plants by Damaging Root Cells and Impairing Soil Nitrogen Cycling. J. Hazard Mater. 2023, 443, 130384 10.1016/j.jhazmat.2022.130384. 36444071

[ref114] Le L.-T.; Nguyen K.-Q. N.; Nguyen P.-T.; Duong H. C.; Bui X.-T.; Hoang N. B.; Nghiem L. D. Microfibers in Laundry Wastewater: Problem and Solution. Science of The Total Environment 2022, 852, 158412 10.1016/j.scitotenv.2022.158412. 36055511

